# The link between social communication and mental health from childhood to young adulthood: A systematic review

**DOI:** 10.3389/fpsyt.2022.944815

**Published:** 2022-10-06

**Authors:** Magdalena Dall, Johannes Fellinger, Daniel Holzinger

**Affiliations:** ^1^Research Institute for Developmental Medicine, Johannes Kepler University Linz, Linz, Austria; ^2^Institute of Neurology of Senses and Language, Hospital of St. John of God, Linz, Austria; ^3^Clinical Department of Social Psychiatry, University Clinic for Psychiatry and Psychotherapy, Medical University of Vienna, Vienna, Austria; ^4^Faculty of Humanities, Institute of Linguistics, University of Graz, Graz, Austria

**Keywords:** social communication, pragmatic language, mental health, depression, conduct problems, peer problems

## Abstract

This systematic review investigates the current state of the literature on the association between social communication (SC) skills and mental health outcomes in children and young adults. We ran searches using terms describing SC and mental health in the following databases: PubMed, the Psychology and Behavioral Sciences Collection, APA PsychInfo, the Education Resource Information Center (ERIC) and the Cumulative Index to Nursing and Allied Health Literature (CINAHL). We included studies that both measured SC before age 21 years and assessed a mental health component, such as behavior/conduct problems, anxiety, depression, or emotional problems, before age 30. Only peer-reviewed articles published in or after 2000 were retained. In total, 27 studies were included in the narrative synthesis. Overall, the methodological quality was good. A longitudinal design was used in 12 studies, seven of which sourced data from the Avon Longitudinal Study of Parents and Children. Most studies investigated the broad concept of pragmatic language and its influence on internalizing, externalizing, and peer-relationship problems. The analyzed publications included population-based studies and studies investigating special populations with existing mental health problems or at increased risk of developing them. The population-based cross-sectional studies showed significantly higher rates of SC problems in children with mental health problems while those with a longitudinal design found earlier onset and more persistent mental health problems in children who had the most severe SC difficulties. Studies with special populations confirmed more SC problems as compared to typically developing children and worse mental health outcomes in children with additional SC difficulties. The available literature thus shows consistently significant, mostly moderate associations between SC skills and mental health outcomes. SC difficulties earlier in life can predict mental health problems and impact their trajectories.

**Systematic review registration:**

[https://www.crd.york.ac.uk/prospero/display_record.php?RecordID=286598], identifier [CRD42022286598].

## Introduction

Social communication (SC) is the appropriate use and interpretation of verbal and non-verbal signals in a social context to connect with other people. SC skills include communicative functions (e.g., making requests, directing somebody’s attention, apologizing, and arguing); conversational skills, such as staying on topic, taking turns, and repairing communication breakdowns; and the adjustment of means of communication to the current situation and interlocutor (e.g., to his/her interests and prior knowledge) (1). Although the terms “pragmatics” and SC are often used interchangeably, SC is considered a broader concept, including verbal and non-verbal communication and with a rather functional definition, whereas pragmatics is more concerned with the use of linguistic rules in verbal interaction that make language relevant in context and appropriate to social interactions, typically not including non-verbal communication (2).

Mental health problems in children negatively impact all dimensions of childhood development, and are particularly linked to emotional and behavioral problems. The most common mental health problems in children include internalizing problems, such as anxiety disorders and depression, and externalizing problems, such as conduct disorder and hyperkinetic disorder (3, 4). In a recent meta-analysis including 61,545 children aged 4–18 years in high-income countries, the prevalence rate of any mental disorder was 12.7%; the prevalence rates for individual disorders included 5.2% for any anxiety disorder, 3.7% for attention deficit hyperactivity disorder (ADHD), 3.3% for oppositional defiant disorder, 1.3% for conduct disorder, and 1.3% for major depressive disorder (5). We are interested in exploring the role of SC deficits for mental health, as SC deficits have been shown to respond to intervention (6).

In a meta-analysis of 22 studies investigating underlying language difficulties in children with emotional and behavioral disorders, both expressive and receptive language skills were found to be below average (7). Further studies have shown that language impairment could be related to peer-relationship problems (8), externalizing behavior (9), and internalizing behavior (10). The question arises whether SC skills have a stronger influence on mental health outcomes than structural language skills, thus pointing to the importance of assessing SC skills in children with mental health problems.

This systematic review aims to evaluate the current state of the literature on the relationship between SC difficulties and mental health outcomes in children, adolescents and young adults. Specifically, we tackle three research questions:

1) What are the associations between concurrent SC difficulties and mental health problems:
  a. In the total population?
  b. In groups of children with mental health conditions?
2) Generally, do previously diagnosed SC difficulties predict mental health problems later in life?
3) How are SC difficulties associated with the developmental trajectories of specific mental health conditions?

## Materials and methods

This study was registered on the international PROSPEctive Register Of systematic reviews (PROSPERO) in January 2022 (CRD42022286598).

### Search strategy

**FIGURE 1:**
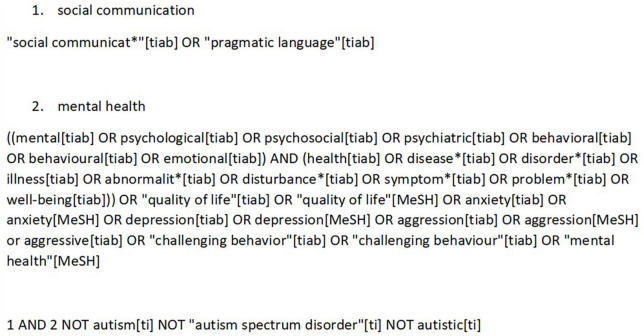
Search strategy PubMed.

The following databases were searched: PubMed, the Psychology and Behavioral Sciences Collection, APA PsychInfo, the Education Resource Information Center (ERIC), and the Cumulative Index to Nursing and Allied Health Literature (CINAHL). The last four databases were searched using the EBSCOhost database. The search strategy was adapted to PubMed and EBSCOhost, using database-specific search terms. As an example, the search string for PubMed is presented in Figure 1. The search string for EBSCOhost can be found in Supplementary 1. Only peer-reviewed papers were included in this systematic review; we did not consider gray literature. The searches were last run on December 23, 2021. The reference lists of all papers included in the full-text-review stage were searched for additional eligible articles. The searches were restricted to studies published in or after 2000. Only studies published in English or German were included.

### Selection criteria

#### Title–abstract review

The exclusion criteria for this first selection phase were: (a) publication in a language other than English or German; (b) publication before the year 2000; (c) case series with less than 10 participants, case studies, systematic reviews or meta-analyses; (d) SC not measured before the age of 21 or not replicable; (e) mental health not measured before the age of 30 or not measured with standardized instruments; (f) inclusion of participants with neurological or other psychiatric disorders, such as intellectual disability, schizophrenia, traumatic brain injury, multiple sclerosis, epilepsy and autism spectrum disorder (ASD); (g) no hint, that a relationship between SC and mental health was explored. If the age at which SC and/or mental health was measured was unclear or not mentioned within the abstract, the paper still progressed to the next selection stage. The first 150 abstracts (22% of the total) were independently double-screened by the first and third authors. The initial kappa for interrater reliability was 0.61. All disagreements were resolved by consensus. Afterward, 100 more abstracts were independently double-screened and the kappa improved to 0.7, which is considered substantial (11). Subsequently, the first author finished the screening of the remaining abstracts.

#### Full-text review

Full texts of all studies that passed the title–abstract review were retrieved and read by the first author, who applied the same exclusion criteria as in the previous selection stage. At this stage, all publications were examined in more detail, especially those whose abstract was unclear on or missing information relevant to our exclusion criteria. If the first author was unsure about a study, the last author read the study as well and the final decision on including each study was made jointly by the first and third authors.

### Description of the variables

All three authors agreed on the variables for which relevant data should be extracted: study characteristics, SC (independent variable), mental health (dependent variable), and confounders. The SC construct varied between publications, and the variable investigated mostly depended on the instrument used to assess SC. We expected most studies to have investigated the broader concept of pragmatic language skills, usually assessed by comprehensive and multidimensional instruments, as well as more specific aspects of SC such as conversational skills, non-verbal communication (including joint attention and early reciprocity), and comprehension of humor and irony. With the search strings used for this systematic review we assume that children with the diagnoses of social (pragmatic) communication disorder (DSM-5) or developmental language disorder with impairment of mainly pragmatic language (ICD-11) are included in the studies. However, due to rare use of these diagnoses in clinical practice and research, and due to an assumed lack of precision regarding the differentiation from language and mental health conditions, we decided not to use the clinical diagnoses in this systematic review but to focus on social communication symptoms. Regarding the mental health outcomes, data were extracted on conduct problems, hyperactivity/inattention, emotional difficulties including symptoms of anxiety and depression, and peer-relationship problems. The following confounders were also preselected: sex, socio-economic status, ethnicity, IQ, multilingualism, age, comorbidities, structural language, and education.

### Methodological quality

All publications were assessed for their methodological quality. For this purpose, we adjusted the screening instrument proposed by Chacón-Moscoso et al. (12) to include the relevant information for this systematic review. Specifically, the following criteria were rated: (a) whether inclusion and exclusion criteria were mentioned; (b) what methodology was used; (c) for longitudinal studies only, whether the attrition rate was mentioned, (d) also for longitudinal studies only, whether the outcome was measured concurrently with or later than the independent variable; (e) whether all outcome variables measured in t_1_ also were measured in t_2_; (f) whether standardized instruments were used to measure the outcome variable/s of mental health; (g) whether a standardized instrument was used to assess the independent variable of SC; (h) whether SC and mental health were assessed by different people and, if so, whether blinding was present (e.g., one instrument parent-reported and another teacher-reported, or directly assessed); (i) how the mental health construct was defined; (j) how the SC construct was defined; (k) whether the responder rate is clearly mentioned; (l) whether representativeness of the sample is mentioned; (m) if there was attrition during the study period, whether missing data were statistically imputed; and (n) whether the study design was prospective or retrospective. The methodological quality assessment was not a reason to exclude any of the papers.

### Data extraction

The data-extraction form was piloted by the first and third authors on the first five publications. Differences were discussed and agreements reached. Additionally, the data-extraction variables were slightly adjusted to include all relevant data. Subsequently, all papers were independently double-coded by the first and third authors. Interrater reliability was assessed and all differences were marked and discussed until agreement was reached. For publications that reported both cross-sectional and longitudinal studies, only data for the longitudinal study were extracted.

Interrater reliability of the methodological quality assessment was acceptable for all items, with one item above 70%, six items above 80% and seven items above 90% agreement.

## Results

### Study selection

**FIGURE 2:**
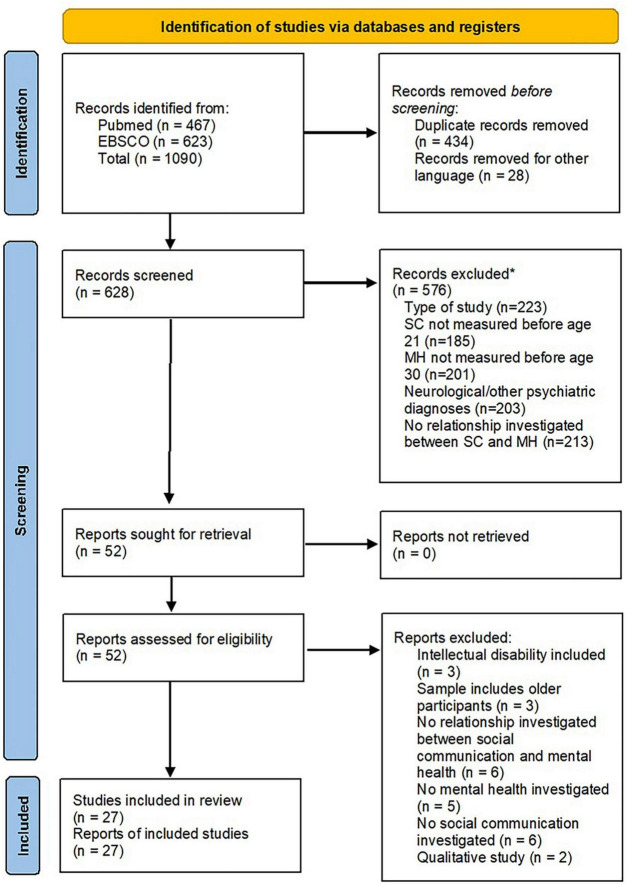
Identification of studies of via databases and registers. *Publications could be excluded for more than one reason (13).

The search strategy yielded an initial total of 1,090 publications. After removing duplicates and papers written in a language other than English or German, 628 publications progressed to the first screening stage. The title–abstract review eliminated 576 publications, leaving 52 publications for the full-text review. After this last stage, 27 publications were retained for data extraction and review (see Figure 2).

### Methodological quality

All 27 studies presented clear inclusion and exclusion criteria. A total of 12 studies (44.4%) had a longitudinal design: ten of these studies mentioned attrition (83.3%), while eight (66.7%) measured mental health at all time points; eight (66.7%) measured the independent and dependent variables concurrently, while the other four (33.3%) measured the outcome variable only after measuring the independent variable. The independent variable was measured with a standardized instrument in 23 studies (85.2%), while the dependent variables were measured with standardized instruments in 24 studies (88.9%). Control techniques, such as different informants for the dependent and independent variables, were reported in nine studies (33.3%). The construct definitions of the mental health variables and SC variables were replicable in all studies (100%). Twenty studies (74.1%) mentioned the responder rate, and the study samples were representative of the total population in 14 studies (51.9%). Only three papers (11.1%) reported that missing data were imputed, even though ten studies mentioned attrition. All 27 studies had a prospective study design (see Supplementary 2 for detailed information).

### Study characteristics

Of the 27 reviewed studies, seven drew participants from the Avon Longitudinal Study of Parents and Children (ALSPAC) database. As these studies tackle different research questions, they each include a different number of individuals, with high degrees of overlap in their samples. Additionally, two publications included participants from the Manchester Language Study. Therefore, 20 different study samples were included in our review. Most of the studies were conducted in Europe [20]; five were carried out in North America (United States and Canada) and the other two in Australia. A complete lack of studies from South America, Africa, and Asia should be noted. It is evident from the publication dates that SC has gained increased interest over recent years. Since 2015, 14 studies have been published on SC and the mental health outcomes within the scope of this review, compared to just 13 in the preceding 15 years. Twelve studies explored the associations between SC and mental health with a longitudinal design, whereas the other 15 studies used cross-sectional data. In terms of sampling, two types of studies were identified: twelve population-based studies drew participants from a large population of children, applying no exclusion criteria; the other 15 studies investigated special populations, such as children with neurodevelopmental disorders like ADHD or with specific language impairments. Six of those 19 studies also included a control group. Regarding age focus, four studies assessed SC in preschool children (<7 years), 22 studies measured SC in children between 7 and 15 years old, and one study assessed SC in adolescents (>15 years). The mental health outcome variable was assessed in preschool children in four studies, in children between 7 and 15 years in 20 studies, and in adolescents above the age of 15 years in three studies.

### Instruments and measures

This section outlines the most commonly used instruments for assessing SC and mental health in the reviewed studies. All other instruments are mostly used in just one study and will be mentioned within the narrative synthesis.

The most commonly used standardized instrument to assess SC is the original Children’s Communication Checklist (CCC) (14) or its second edition, CCC-2 (15). The CCC is typically parent or teacher reported. The CCC can be used to calculate a pragmatic language composite score, combining scores on five subscales: inappropriate initiation; coherence; stereotyped conversation; context and rapport. Lower scores indicate greater impairment. Most studies used the cut-off value of ≤140 corresponding to scores below the 10th percentile for the pragmatic composite score.

The CCC-2 (15) includes ten subscales: speech, syntax, semantics, coherence, inappropriate initiation, stereotyped language, use of context, non-verbal communication, social relations, and interests. The CCC-2 is typically completed either by parents or teachers. Unlike for the CCC, higher scores indicate greater impairment. The standardized pragmatic language composite score is not available anymore in the CCC-2; instead, a score below the tenth percentile on any of the subscales might indicate an impairment. Nevertheless, several studies have used the CCC-2 to calculate a pragmatic language composite score by summing scores on the same five subscales used in the CCC. In total, these two instruments were used in 17 studies.

The Social and Communication Disorders Checklist (SCDC) (16) is a standardized screening instrument completed by parents to assess autistic traits in children. It includes 12 statements about the child and gives three response options: “not true,” “quite or sometimes true,” and “very or often true”.

The Social Communication Questionnaire (17) is a standardized screening instrument for ASD and includes 40 yes/no questions for parents to answer about their children. It comprises three subscales: reciprocal social interaction, communication, and repetitive behaviors. A total score ≥15 indicates a likelihood of ASD. The questionnaire has high reliability (0.84–0.93) and a good internal consistency with a Cronbach’s Alpha of 0.86.

To measure mental health, 13 studies used the Strengths and Difficulties Questionnaire (SDQ) (18), which is a well-established standardized behavioral screening instrument, filled in by either parents or teachers. It includes five dimensions: emotional symptoms, conduct problems, hyperactivity/inattention, peer-relationship problems, and prosocial behavior. Additionally, a total difficulties score can be determined: a total score of 13–15 shows slightly raised problems (borderline range: 10th–20th percentile), while a score ≥16 indicates high or very high problems (below the 10th percentile).

The three studies that analyzed depressive symptoms as the outcome variable used the full Moods and Feelings Questionnaire (19) or its short version (20). This is a standardized screening tool for depression in children and adolescents aged 6 to 19 years, and has two versions: child-reported and parent-reported. The instrument comprises 33 phrases describing how the person has been feeling or acting over the past 2 weeks. Higher scores point to more severe depressive symptoms. The maximum score on the long [short] version is 66 [26], and a score ≥27 [12] may indicate the presence of depression.

The Conners 3rd Edition Scales are a standardized multi informant rating scale for parents, teachers (6–18 years) and children and adolescents themselves (8–18 years) to assess behavior with a focus on ADHD and it’s most common co-morbidities (21).

### Narrative synthesis

Twelve of the studies are population-based, while the other 15 investigate special populations of children and adolescents either with already established mental health problems (emotional and behavioral problems) or with increased risk of developing mental health problems, such as socially disadvantaged children (detailed information on all studies included can be found in Supplementary 3). Available research on special populations of children with mental health problems reports cross-sectionally on their SC skills as compared to typically developing children, correlations with negative mental health outcomes in case of comorbid SC difficulties or describes the association between SC skills and the development of mental health symptoms over time. In the following, we first describe the characteristics of population-based studies, considering cross-sectional (Table 1) and then longitudinal studies (Table 2), before turning to the special-population studies, again separately considering cross-sectional (Table 3) and longitudinal studies (Table 4). All studies are sorted according to their publication date, except for Table 2, where all ALSPAC studies are listed consecutively, followed by the other studies.

**TABLE 1:** Characteristics of population-based cross-sectional studies.

Population based studies							
Cross-sectional							
Author/s	Year	Country	N	Age when SC measured	Age when mental health measured	SC measure/s	Results
Möricke et al. (22)	2013	The Netherlands	6,330	14.84 (SD = 2.19) months	14.84 (SD = 2.19) months	Utrecht Screening Questionnaire based on factor analysis (non-stand.; par.)	Latent class analysis identified a latent class (5.7%) with high scores on non-typical communication, negative emotionality, demanding behavior, social anxiety, advanced social interaction problems, and sleep problems; another latent class (16.4%) had high scores on non-typical communication and specific problems in language and communication.
Ketelaars et al. (24)	2010	The Netherlands	1,364	4.11 years (SD = 4 months)	4.11 years (SD = 4 months)	CCC (stand.; teach.)	Children with pragmatic language impairment scored two to three times the sample mean on all subscales of the SDQ and had a risk ratio for high/very high difficulties (total score) of 11.3 for boys and 12.3 for girls. In a regression analysis, structural language explained 14.8% of total problem behavior. After adding pragmatic competence and autistic behavior, the model explained 61.6% of the SDQ total difficulties score. Pragmatic competence was the most important predictor.
Theriault et al. (25)	2001	Canada	157	19.78 years	19.78 years	Pragmatic Language Inventory (Non-stand.; self)	The lack of pragmatic language abilities (mostly the presence of verbal impulsivity) almost completely explained the relationship between ADHD symptoms and relationship aggression.

ADHD, attention deficit hyperactivity disorder; CCC, Children’s Communication Checklist; non-stand., non-standardized; par., parent reported; SDQ, Strengths and Difficulties Questionnaire; self, self-assessment; stand., standardized; teach., teacher reported.

**TABLE 2:** Characteristics of population-based longitudinal studies.

Population based studies							
Longitudinal							
Author/s	Year	Country	N	Age when SC measured	Age when mental health measured	SC measure/s	Results
Rice et al. (26)	2019	England	7,543	7 years	10.64 (SD = 0.25) and 18.65 (SD = 0.49)	CCC (stand.; par.) SCDC (stand.; par.)	Significantly higher rates of childhood SC and pragmatic language problems were identified in individuals with early adolescence onset of depression, compared to those with later adolescence onset (SC: OR = 0.68; Pragmatic difficulties: OR = 1.31)
Sullivan et al. (28)	2017	England	7,058	7, 10, 13, 16 years	12 and 18	SCDC (stand.; par.)	SC abilities at ages 7 and 10 were significantly associated with depressive symptoms at 12 years (OR = 1.22), and SC abilities at ages 13 and 16 were significantly associated with depressive symptoms at 18 years (OR = 1.21), even after controlling for sociodemographic variables (including IQ) and autism traits.
Sullivan et al. (27)	2016	England	7,659	9 years	12 and 18	CCC (stand.; par.)	Poorer pragmatic language at age 9 was associated with depression at age 18 (OR = 1.10). Poorer expressive speech and language abilities were not associated with depression at either age.
Riglin et al. (29)	2016	England	6,664	7–9 years	3.9, 6.8, 8.1, 9.6, 11.7, 13.1, and 16.5 years	CCC (stand.; par.) SCDC (stand.; par.)	The persistent ADHD trajectory from 4 to 17 years was significantly associated with multimorbidity: within this group 53.1% had SC problems, 27.8% had pragmatic language impairments, 24.1% had low IQ, and 42% had conduct problems in childhood.
Law et al. (30)	2015	England	2,915	9 years	13 years	CCC (stand.; par.)	Pragmatic language skills partly mediated the association between social disadvantage and adolescent behavior (emotional and behavioral) in middle childhood, even after controlling for sex, age, and IQ.
Oliver et al. (31)	2011	England	6,074	9 years	4, 7, 8, 10, 12, and 13 years	CCC (stand.; par.) SCDC (stand.; par.)	SC difficulties were significantly greater in all conduct-problems groups, particularly in the early onset-persistent group. Controlling for demographic confounders and child verbal IQ did not change the associations.
Skuse et al. (32)	2009	England	8,094	7.8 years	8.4 years (SD = 3.7 months)	SCDC (stand.; par.)	SC difficulties were significantly associated with all SDQ behavior outcomes about 1 year later, after controlling for sex, IQ, and maternal education.
Askeland et al. (33)	2021	Norway	15,205	0.6, 1.5, 3, and 8 years	1.5, 3, 5, and 8 years	Ages and stages SC (34) (stand.; par.)M-CHAT (35), (stand.; par.) SCQ (36) (stand.; par.), short version CCC-2 (stand.; par.)	SC at age 5 correlated moderately with hyperactivity symptoms at age 8.
Beernink et al. (37)	2007	The Netherlands	1,803	14 and 19 months	14 and 19 months	Self-constructed questionnaire (non-stand.; par.)	The variable “communicative intent” at 14 months correlated significantly with oppositional behavior and attention at 19 months.

ADHD, attention deficit hyperactivity disorder; CCC, Children’s Communication Checklist; CCC-2, Children’s Communication Checklist 2nd Edition; M-CHAT, Modified Checklist for Autism in Toddlers; non-stand., non-standardized; OR, odds ratio; par., parent reported; SC, Social Communication; SCDC, Social and Communication Disorders Checklist; SCQ, Social Communication Questionnaire; stand., standardized; teach., teacher reported.

**TABLE 3:** Characteristics of special population cross-sectional studies.

“Special populations”								
Cross-sectional								
Author	Year	Country	N	Age when SC measured	Age when mental health measured	Special population	SC measure	Results
Eadie et al. (38)	2021	Australia	41	9.4 (SD = 1.6) years	9.4 (SD = 1.6) years	Social, emotional and behavioral difficulties	CCC-2 (stand.; teach)	In the small sample of 15 measured with the teacher version of the CCC-2, no significant correlation was found between the Social Interaction Difference Index Score and any SDQ score.
Parke et al. (39)	2021	United States	50	10.57 (SD = 2.09) years	10.57 (SD = 2.09) years	ADHD	CCC-2 (stand.; par.)	Pragmatic language skills were significantly correlated with externalizing behavior (0.59) and behavioral symptoms of ADHD (0.74), and with declines in adaptive skills (−0.74), even after controlling for social cognition. Pragmatic language and cognitive empathy explained a substantial amount of variance in problematic and adaptive behaviors (*R*^2^ = 0.51).
James et al. (40)	2020	Australia	54	13.03 years	13.03 years	Emotional and behavioral difficulties	ERRNI (41) (stand.; dir.) NSS (42) (stand.; dir.)	Individuals with emotional and behavioral difficulties scored significantly lower on narrative skills and SC compared to typically developing students. In the full sample, SC was found to be significantly correlated with SDQ emotional (−0.422), SDQ peer problems (−0.293), SDQ externalizing behavior (−0.462), Conners-3 aggression (−0.354), and Conners-3 peer relationships (−0.386).
Mewhort-Buist and Nilsen (43)	2019	Canada	169	10.6 years (SD = 6 months)	10.6 years (SD = 6 months)	Shyness	12 story scenarios vignettes (44) (non-stand.; dir.)	In shyer children, better irony comprehension was related to increased loneliness (β = 0.581) and depression symptoms (β = 0.678), and to fewer prosocial peer experiences (β = −0.460).
Brenne and Riemehau (45)	2019	Norway	73	8–13 years	8–13 years	Outpatient population of child psychiatric clinic	CCC (stand.; par.; teach)	Children with parent-reported pragmatic language impairment had significantly higher symptom scores on four Child Behavior Checklist scales: Anxious/depressed, withdrawn/depressed, social problems, and thought problems. Pragmatic language correlated significantly with the domains anxious/depressed (0.34), withdrawn/depressed (0.32), social problems (0.47), thought problems (0.38), and attention problems (0.20).
Hollo et al. (46)	2019	United States	46	12.2 (SD = 2.5) years	12.2 (SD = 2.5) years	Emotional disturbance	CASL pragmatics (47) (stand.; dir.)	Pragmatic scores were lower in all subgroups of emotional or behavioral difficulties (internalizing, externalizing, and both) than the scores for receptive and expressive language. The internalizing group was superior in expressive, receptive language, and pragmatics, with the lowest scores in pragmatics.
Halls et al. (48)	2015	England	404	10.13 (SD = 1.6) years	10.13 (SD = 1.6) years	Social Anxiety Disorder	SCQ (stand.; par.)	Significantly higher deficits across all SC domains (social interaction, communication) were found in children with social anxiety disorders, as compared to other anxiety disorders.
Roy and Chiat (49)	2014	England	91	10.5 years (SD = 6.74 months)	2.5–4; 4–5; and 10.5 years	Language difficulties	Social Responsiveness Scale (50) (stand.; par.)	All children with SC problems (combined with language difficulties or not) had higher SDQ total difficulties scores than those without SC difficulties. SC scores and SDQ scores were significantly correlated (0.78).
Law et al. (51)	2014	Scotland	138	8.9 (SD = 1.9) years	8.9 (SD = 1.9) years	Socially disadvantaged background	CCC-2 (stand.; teach)	Pragmatic difficulties mediated the effects of language skills on emotional problems (85.1% mediated), peer problems (87%), and the SDQ total difficulties score (66%) after controlling for IQ and sex.
Mackie and Law (52)	2014	Scotland	77	10.12 years	10.12 years	Emotional/behavioral difficulties	CCC-2 (stand.; teach)	Significantly lower CCC-2 composite and subscale scores were found for the group of boys with externalizing behavior, relative to the control group.
Nilsen et al. (53)	2013	Canada	53	8.01 (SD = 1.1) years	8.01 (SD = 1.1) years	ADHD	CCC-2 (stand.; par.)	Pragmatic impairment was found to be significantly correlated with ADHD symptoms, inattention (0.83), hyperactivity/impulsivity (0.79), and executive functioning (0.75), even after controlling for child’s age and receptive vocabulary.
Gilmour et al. (54)	2004	England	55	10.2 years (SD = 2.7 months)	10.2 years (SD = 2.7 months)	Conduct or pervasive developmental disorders	CCC (stand.; par.)	Of the children with conduct disorder, two-thirds had very high rates of SC problems (in the clinical range)—a comparable rate to that of children with ASD. SC was not significantly related to Verbal IQ or Performance IQ.

ADHD, attention deficit hyperactivity disorder; ASD, Autism Spectrum Disorder; CASL, Comprehensive Assessment of Spoken Language; CCC, Children’s Communication Checklist; CCC-2, Children’s Communication Checklist 2nd Edition; dir., direct assessment; non-stand., non-standardized; NSS, Narrative Scoring Scheme; par., parent reported; self, self-assessment; SC, Social Communication; SCQ, Social Communication Disorder; SDQ, Strengths and Difficulties Questionnaire; stand., standardized; teach., teacher reported.

**TABLE 4:** Characteristics of special population longitudinal studies.

“Special populations”								
Longitudinal								
Author/s	Year	Country	N	Age when SC measured	Age when mental health measured	Special population	SC measure/s	Results
Conti-Ramsden et al. (55)	2019	England	168	11 years	7, 8, 11, and 16 years	Language Impairment	CCC (stand.; teach)	Significantly poorer pragmatic language abilities were found in the group with childhood-onset-persistent emotional and peer-relationship problems, as compared to the resolving emotional group and low-level group.
Mok et al. (56)	2014	England	171	7 and 11 years	7, 8, 11, and 16 years	History of language impairment	CCC (stand.; teach)	Pragmatic language difficulties at age 7 were 2.5 times more likely for children with childhood-onset persistent peer problems at age 11 than for those with low/no peer problems.
Helland et al. (57)	2014	Norway	40	13.47 (SD = 0.82) years	7–9 and 13.47 (SD = 0.82) years	Behavioral difficulties	CCC-2 (stand.; par.)	Pragmatic language ability at age 12–15 years correlated significantly with the pragmatic language composite score (*r* = −0.52), with the SDQ subscales emotional problems (*r* = −0.40) and peer problems (*r* = −0.048), and with the SDQ total difficulties score (*r* = −0.05) at age 7–9 years. In a multiple regression model, language and peer problems at t_1_ significantly predicted pragmatics at t_2_ (*R*^2^ = 36%).

CCC, Children’s Communication Checklist; CCC-2, Children’s Communication Checklist 2nd Edition; dir., direct assessment; non-stand., non-standardized; par., parent reported; SDQ, Strengths and Difficulties Questionnaire; self, self-assessment; stand., standardized; teach., teacher reported.

#### Population-based studies

##### Cross-sectional studies

The first (22) of three cross-sectional population-based studies (Table 1) examined empirically derived developmental profiles (social-communicative problems, internalizing and externalizing behaviors) in a large sample of infants (*n* = 6,330; age 14–15 months) in the Netherlands. It used a compilation of items from standardized parent questionnaires suitable for children younger than 18 months. Exploratory factor analysis revealed nine factors, and latent class analysis yielded five classes. Besides two classes with relatively typical behaviors (67.1%) and one class with symptoms of regulation disorder (10.8%), two classes had communication and/or social-interaction problems. The first (5.7%) included children with severe communication and social-interaction problems, characterized by non-typical early non-verbal and verbal communication and primarily internalizing problems (social anxiety/inhibition, negative emotionality, sleep problems)—a profile that resembles multi-system developmental disorder (23). The other class with communication problems (16.4%) had slightly less severe problems in communication, language, and speech development, and otherwise rather typical behaviors (similar to the first two classes).

In the second cross-sectional population-based study, Ketelaars et al. (24) examined the incidence of emotional and behavioral problems in 1,364 4-year-old children in the Netherlands using teacher-reports (CCC and SDQ). Highly significant partial correlations were found between the pragmatic composite score of the CCC (controlling for sex) and the SDQ total difficulties score (−0.69), hyperactivity/inattention (−0.60), peer-relationship problems (−0.43), conduct problems (−0.37), and emotional problems (−0.33). On all SDQ problem subscales, children with pragmatic language impairment (defined by a CCC score ≤132) showed highly elevated scores (two to three times the sample mean). Boys exhibited lower pragmatic skills and, consequently, higher rates of emotional and behavioral problems. Notably, structural language scores did not correlate with mental health problems: in children with speech and syntax problems, an average probability of emotional and behavioral problems was found. In a regression analysis, pragmatic competence was the most important predictor of total problem behavior.

In the last cross-sectional population-based study (25), the link between ADHD symptoms and relationship aggression in 157 college students could almost completely be explained by verbal impulsivity (1 of 4 factors resulting from a principal component analysis of the 22 items of the pragmatic language inventory). Participants who were verbally impulsive were more likely to engage in relationship aggression.

Even though the three population-based cross-sectional studies investigated different age groups (ranging from 14 months to 19 years), all of them reported an association between SC problems and behavioral issues. While only one study (24) used standardized instruments to assess SC and emotional and behavioral problems, the other two studies (22, 25) used the results of factor analysis from non-standardized parent questionnaires and a non-standardized self-reported questionnaire. The two larger studies (22, 24) were both conducted in the Netherlands, the last study (25) with only 157 participants in Canada. Examining precursors of child psychiatric disorders in a large sample in early toddlerhood (<1.5 years) it was found that communication and language problems did not necessarily co-occur with mental health problems. However, in case of additional advanced social interaction problems (comparable to multi-system disorders) they were associated with internalizing problems. The class with severe communication and social-interaction problems was significantly older than the remaining sample, pointing toward a more differentiated profile with age. When looking at children slightly older (24), at the age of 4 years, pragmatic language impairment rather than structural language skills were moderately to strongly correlated with emotional and behavioral problems. The last study only included a small sample of college students and focusses on a very specific research question of the correlation between ADHD and aggression in relationships, which was almost completely explained by verbal impulsivity (pragmatic language difficulties) rather than other aspects of ADHD.

##### Longitudinal studies

Seven of the nine population-based longitudinal studies (Table 2) analyze ALSPAC data, collected from a large representative sample of children born during 1991–1992.

Rice et al. (26) analyzed the relationship between SC and mental health by examining the shapes of depression trajectories between the age of 10.6 and 18.7 years. They identified three trajectory classes: persistently low (73.7%), early adolescence onset (9.0%), and later-adolescence onset (17.3%). Pragmatic language difficulties (measured with the CCC) and SC difficulties (measured with the SCDC) at age 7 showed significantly higher incidences in the early adolescence-onset class than in the persistently low risk class (OR = 0.63 and OR = 1.50, respectively). Likewise, pragmatic language difficulties showed a significantly higher incidence in the later-adolescence-onset class than in the persistently low risk class (OR = 0.82). In addition, a comparison between the early- and later-adolescence-onset classes showed significantly higher incidences of pragmatic language and SC difficulties in the former group (OR = 1.31 and OR = 0.60, respectively).

Sullivan et al. (27, 28) reported significant associations between SC problems in mid-childhood and depressive symptoms in adolescence. The more recent study by Sullivan et al. (28) found that poor SC at 7 and 10 years old (mean score) was significantly associated with depression at age 12 (OR = 1.22), and that SC problems at age 13–16 (mean scores) were associated with depressive symptoms at age 18 (OR = 1.21). Associations with depressive symptoms at both ages remained after controlling for intelligence and autistic traits. Moreover, poorer pragmatic skills at 9 years (assessed by the parent-rated CCC) was associated with depression at 18 years (OR = 1.10), even after controlling for sex, maternal education, marital status, and non-verbal IQ. Their first study (27) reported a significant association between SC at age 9 and depressive symptoms at age 18 (OR = 1.10); however, there was no significant association between SC at age 9 and mental health at age 12. In both studies, SC was assessed by parents using the Social Communication Disorders Checklist (16), whereas the adolescents self-reported depressive symptoms. Both studies demonstrated that pragmatic language deficits precede depressive symptoms in late adolescence.

Another ALSPAC study (29) examined ADHD symptom trajectories from age 4 to 17 and correlations with neurodevelopmental traits including SC problems (measured with the SCDC) and impairment of pragmatic language (measured with the CCC). Latent class analysis produced four classes of ADHD trajectories: low (82.6%), intermediate (7.7%), childhood-limited (5.8%), and persistent (3–9%). SC and pragmatic language impairments were found most often in the persistent-trajectory group (53.1 and 27.8%), followed in turn by the groups whose trajectory was childhood-limited (22.9 and 10.3%), intermediate (19.9 and 7.7%), and low (3.4 and 1.2%).

Law et al. (30) investigated the relationship between early disadvantage, pragmatic skills (measured with the CCC), and mental health outcomes (measured with the SDQ). Pragmatic skills at age 9 were found to play a major role in mediating the relationship between social disadvantage and all problem domains of the SDQ (emotional, conduct, hyperactivity, and peer-relationship problems) at age 13, even after controlling for verbal and non-verbal IQ and sex. Pragmatic language skills thus contribute to positive mental health, particularly in children from more socially disadvantaged backgrounds.

In an earlier study of the ALSPAC cohort, Oliver et al. (31) examined associations between trajectories of conduct problems through childhood to adolescence (4–13 years) and social-cognitive competencies. Four conduct-problem pathways were identified: early onset persistent (9.0%), childhood-limited (9.0%), adolescent-onset (14.7%), and low (64.6%). SC difficulties (as measured with the CCC and SCDC) were significantly greater in all conduct-problem groups, with deficits in the early onset-persistent group especially marked. The associations between SC difficulties and conduct disorders remained robust after controlling for demographic confounders, verbal IQ, and internalizing and inattention symptoms. For boys, overactivity symptoms were found to overlap with SC deficits.

The last study of the ALSPAC cohort (32), investigated SC deficits at the age of 7 years 8 months (measured with the SCDC) and found a small but highly significant association with teacher-reported peer-relationship problems, hyperactivity, and conduct problems, and a moderate association (*r* = 0.405) with the SDQ total difficulties score at age 8 years 4 months. Interestingly, elevated verbal IQ was found to act as a protective factor with respect to SC impairment in girls but not in boys.

A Norwegian study (33) of 15,205 children from a longitudinal birth cohort investigated the associations of polygenic risk for ADHD, autism, and schizophrenia with neurodevelopmental traits including SC difficulties. Although testing correlations between earlier SC scores and mental health outcomes was not the study’s primary objective, it found moderate correlations between SC scores at age 5 and hyperactivity symptoms at age 8.

The last longitudinal population-based study (37) examined the correlations between SC at age 14 months and emotional, attentional, and impulsive behavior at 19 months, using assessments by parents of 1,803 infants. Principal component analysis yielded seven factors. Significant but low correlations were found between the factor “communicative intent”—including preverbal communicative behaviors such as babbling, sounds of joy, social gestures, and reacting to communicative approaches—at age 14 months and attention, and oppositional behavior at age 19 months. In addition, problems in communicative intent proved to be quite stable between 14 and 19 months (*r* = 0.466).

All longitudinal population-based studies were conducted in Europe, including the seven ALSPAC publications (26–32). All studies included a large sample size, ranging from 1,800 to 15,000 participants. Most studies investigated children around the age of 7–9 years up to adolescents at the age of 18 years. Only two studies included toddlers below the age of 2 years and children up to 8 years. All studies except for one (37) used standardized instruments to assess SC and mental health. Three publications reported trajectory groups for depression (26), ADHD (29), and conduct problems (31). The other studies investigated associations between SC difficulties earlier in life and later depression (27, 28), or with mental health problems in general (30, 32, 37). The three publications looking at trajectories all have in common that the class of persistently low mental health problems showed the least SC and pragmatic language difficulties, while the group with early onset-persistent showed the most difficulties. All other studies consistently demonstrated the contribution of SC difficulties at early school age to the pathogenesis of depression and behavior problems later in life. In very young children, <2 years of age, problems in communicative intent seem to be an aspect of early SC behavior of high relevance for the development of mental health problems. The studies controlling the analysis of effects of SC problems on mental health for demographic confounders, IQ, sex or inattention all reported that their results of significant relationships remained robust. This finding supports the specific character and influence of SC skills.

#### Special-population studies

##### Cross-sectional studies

Twelve of the special-population studies used a cross-sectional design to investigate the relationship between SC problems and mental health symptoms (Table 3). Most of the studies in this category report on SC skills in children and adolescents with emotional or behavioral difficulties, others include children from socially disadvantaged backgrounds, children with language disorders or challenging personality traits such as shyness.

Eadie et al. (38) examined pragmatic language and SC skills in a group of 41 children (6–12 years) with social, emotional, and behavioral difficulties, with or without histories of maltreatment. Only 15 students were measured with the teacher version of the CCC-2, and two-thirds met the criteria for pragmatic language disorder. Students with a suspected history of maltreatment had more severe pragmatic language difficulties as compared to structural language skills. In this small sample, no significant correlations were found between the Social Interaction Difference Index (indicating pragmatic language difficulties that are disproportionally poor in relation to structural language skills) and SDQ dimensions.

Parke et al. (39) assessed pragmatic language and social cognition in 25 children with ADHD and 25 healthy controls (all participants aged 7–13). Children with ADHD performed significantly worse on social cognition (theory of mind, affect recognition, cognitive empathy) and pragmatic language compared to their typically developing peers. In multiple regression analyses to explain problem behaviors, pragmatic language skills (CCC-2) explained a substantial amount of variance in externalizing problems, behavioral symptoms (atypicality, withdrawal, and attention), and adaptive skills, after controlling for IQ and social cognition.

James et al. (40) compared the profiles of narrative skills, social cognition skills (identifying emotions and emotional reactions, understanding social gaffes and conflicting messages), and structural language skills between adolescents attending behavioral support schools (aged 12–16 years) and a comparison group of typically developing students from a mainstream school. Scores for narrative and social cognition skills (both SC) and structural language skills were significantly poorer in the students attending special schools. Only when data were pooled across both groups significant correlations were found between all three skill dimensions (narrative, social-emotional evaluation, structural language) and externalizing behaviors (*r* = −0.389, *r* = −0.462, *r* = −0.304, respectively). In addition, narrative skills rather than language skills were significantly related to emotional problems (−0.365). Social-emotional evaluation scores were significantly related to SDQ scores for emotional (−0.422), peer-relationship (−0.293), and externalizing problems (−0.462), and to increased aggression and peer-relationship problems assessed by the Conners-3 scales.

Mewhort-Buist and Nilsen (43) examined whether relations between shyness and mental health problems (loneliness, depression, peer experiences) were moderated by irony comprehension. Using a series of vignettes and self-report measures of irony comprehension, they found that shyer children with better irony comprehension reported increased loneliness and depression symptoms. Better comprehension strengthened the relationship between shyness and peer victimization for girls but weakened this relationship for boys. This was the only study indicating that for certain populations of children, increased SC skills (irony comprehension) might not be advantageous with regard to their mental health.

Brenne and Rimehaug (45) investigated the association of mental health problems (measured using the Child Behavior Checklist and the Teacher’s Report Form) with pragmatic skills and impairment (measured using the CCC) in a sample of child psychiatric outpatients aged 8 to 13 years (*n* = 73). A high rate of pragmatic language impairment (38% based on parent-reports, 41% based on teacher-reports) was found in the clinical sample. Strong correlations with pragmatic language impairment were reported for not only social problems (0.47) and withdrawal (0.32) but also anxiety (0.34) and rumination (0.38).

Studying a group of 46 boys with emotional or behavioral difficulties (7–17 years old), Hollo et al. (46) found that pragmatic and structural language scores were lower in the externalizing group than in the internalizing group, and were lowest in the group exhibiting both internalizing and externalizing problem behaviors. Pragmatic competencies were lower than the structural language skills in all three subgroups, with the most marked differences between limited pragmatic skills and (typical) language skills in the internalizing group (albeit with a very small sample size, *n* = 8).

Halls et al. (48) studied SC among children with social anxiety disorder (*n* = 262) and anxious children without social anxiety disorder (*n* = 142). Children with social anxiety disorder scored significantly higher on the Social Communication Questionnaire and were three times more likely to score above the clinical cut-offs, compared to anxious children without social anxiety disorder.

Roy and Chiat (49) examined the relations between language skills, SC, and parent-assessed mental health (measured with the SDQ) in children referred as preschoolers to clinical services because of concerns about their language skills (*n* = 91). By 9–11 years old, 27% had language impairment, 11% had SC deficits, and 20% had both. SC deficits were significantly associated with peer problems (OR = 0.55), emotional problems (OR = 0.65), and reduced prosocial behavior (OR = 2.16), after controlling for non-verbal IQ and receptive language skills. By contrast, language impairment was only associated with hyperactivity.

Law et al. (51) focused on 138 children aged 5–12 years from socially disadvantaged backgrounds. Pragmatic difficulties (measured with the CCC-2) were found to mediate the relations of language skills with hyperactivity, peer problems, and the SDQ total difficulties score, even after controlling for IQ.

Mackie and Law (52) investigated structural and pragmatic language skills in a sample of 35 boys (7–12 years) with a high risk of an emotional behavior disorder and 42 controls. As expected, the clinical group scored significantly lower than the control group on all measures of structural language (CELF-4) and pragmatic language (CCC-2). Notably, though, no association was found in the clinical group between language impairment and teacher-rated pragmatic language skills.

Nilsen et al. (53) examined the ability to interpret referential statements, which requires appropriate interpretation of the speaker’s communicative intentions. Comparing 27 children with ADHD (6 to 9 years) and a typically developing comparison group (*n* = 26), they found that children with ADHD made more mistakes in the experimental tasks and that ADHD symptoms were correlated with parent-rated SC scores (CCC-2). Lower accuracy in the interpretations of referential statements might, therefore, contribute to communication difficulties of children with ADHD.

The last cross-sectional study on a special population (54) investigated SC deficits in children aged about 10 years who had conduct disorder (*n* = 55), ASD (*n* = 87), or were typically developing (*n* = 60). 78% of the children with conduct disorder had pragmatic language impairments (parent reported), independent of IQ. This rate is only slightly lower than for children with ASD (89–95%). Only 8% of the typically developing children had pragmatic language impairment. In a second study, including 54 children excluded from elementary schools in socially disadvantaged inner London, about two-thirds of participants had a CCC pragmatic composite score within the clinical range.

The relatively high number of cross-sectional studies on special populations of children with diverse mental health conditions or with high increased risk of mental health problems were mostly based on small samples (between 41 and 404) of children in middle childhood (8–13 years). Only one study (43) used a non-standardized instrument to measure SC. Five (39, 40, 48, 52, 53) of the twelve studies included a control group. The studies were highly diverse with regard to the groups of included children. There were five studies (39, 40, 52–54) looking at externalizing behavior problems, including two studies on children with ADHD. They all found significant associations between pragmatic language difficulties and the mental health condition. The other five studies (38, 43, 45, 46, 48) investigating internalizing behavior problems showed diverse results. Two of these papers found significant associations between SC and social anxiety disorder (48) and emotional and thought problems and social and withdrawal symptoms (45). In a single study (38), no significant correlation between SC and social emotional and behavioral difficulties was found, however the sample only included 15 children. Only one study (43) reported a result in contrast with the others, that better understanding of irony was associated with more internalizing problems. Another study (51) found a mediating role of pragmatics between early disadvantage and peer related problems and hyperactivity in adolescence. Overall, the diverse studies confirmed the increased rate of social communication problems in children with or at risk for mental health conditions and an increase of negative mental health symptoms if SC difficulties co-occurred with established or increased risk for mental health conditions.

##### Longitudinal studies

Finally, we report the three special-population studies with a longitudinal design (Table 4). Two of these three studies explored the developmental pathways of children with language difficulties.

Conti-Ramsden et al. (55) investigated the trajectories of emotional and peer-relationship problems in 168 children with developmental language disorder between the ages of 7 and 16 years. They identified five distinctive trajectories of emotional and peer problems: childhood-onset persistent in both domains (26%), adolescent-onset in both (16%), consistently low in both (11%), emotional problems in childhood only (24%), and increasing peer problems (22%). In contrast to structural language, pragmatic language abilities were significantly associated with group membership (besides child prosocial behavior and parental mental health). Children with a persistent trajectory of high emotional and peer problems from childhood to adolescence had significantly lower pragmatic language scores compared to children in most other groups. The group with increasing peer problems had the lowest pragmatic language competence.

Mok et al. (56) identified four distinct developmental trajectories of peer relations from 7 to 16 years of age in 171 children with a history of specific language impairment: low-level/no problems (22.2%), childhood-limited problems (12.3%), childhood-onset persistent problems (39.2%), and adolescent-onset problems (26.3%). Children with persistent peer problems were 2.5 times more likely to have pragmatic language difficulties, relative to children with low/no problems.

Finally, Helland et al. (57) investigated pragmatic language skills and behavioral problems in a group of 40 children identified as having externalizing behavioral problems at two time points—aged 7–9 years and aged 12–15 years. Compared to a typically developing group, the group with behavioral problems achieved significantly poorer pragmatic scores on 9 of the 10 CCC-2 subscales, with 70% showing a general communication composite score in the clinical range whereas in the comparison group 10.8% showed a general communication composite score in the clinical range. Longitudinal analyses showed that peer and language problems at 7–9 years were significant predictors of pragmatic language impairment in adolescence.

The three studies looking at special populations in a longitudinal design are comparable with regards to the age of the samples (all between 7 and 16 years), and all of them used standardized instruments. The sample sizes varied between 37 and 171 participants. The two studies on children with language disorders (55, 56) included children and adolescents from the Manchester Language Study. Both studies aimed to identify trajectory groups and variables associated with group membership and found persistent peer problems to be related with social communication difficulties. The third study in contrast looked at the longitudinal influence of emotional and peer problems on pragmatic language impairments in adolescence (57). Overall, the studies demonstrate the correlation of SC skills with peer relationships that are again essential for positive mental health, and a bidirectional causality of this relationship.

## Discussion

To the best of our knowledge, this systematic review is the first to summarize the present state of the literature on associations between SC skills and mental health in children to young adults, excluding individuals with ASD. Interest in these associations has evidently been increasing, with a growing number of publications in recent years, particularly in Europe.

From the papers included in this systematic review, it can be concluded that despite the association with other aspects of child development, SC skills are dissociable from non-verbal and verbal intelligence, language, social development, and attention; therefore, SC must be regarded as a self-contained developmental domain. Nonetheless, the acquisition of communication skills involves the dynamic interaction of language, social, and cognitive development (including the development of social cognition, such as theory of mind) (2). Nevertheless, SC skills can be only partly explained by these dimensions. In her study of students with emotional or behavioral disorders, Rinaldi (58) showed that social skills, structural language, and IQ explained only 44% of the variance in pragmatic language skills. Similarly, in Helland et al.’s study (57) of children with emotional and behavioral disorders, only 36% of the variance in pragmatic language problems in adolescence was explained by language and peer-relationship problems at age 7–9 years. Furthermore, associations between SC and mental health cannot be explained by an overlap between the respective measures. The items of the most widely used CCC and CCC-2 domains do not directly relate to mental health symptoms, although some overlap between CCC items and SDQ items measuring peer-relationship problems cannot be excluded.

The methodological quality of the studies was mostly satisfactory and sometimes high, with clear inclusion and exclusion criteria reported in all studies and standardized instruments used to measure the dependent and independent variables in almost 90% of them. Seven of the 12 longitudinal studies are based on the large ALSPAC cohort that permits controlling for confounders. In most of the longitudinal studies, mental health was measured concurrently with and after SC, enabling the impact of earlier mental health problems to be accounted for. Many reviewed studies assessed both SC and mental health with parent questionnaires which might have contributed to the observed associations between the two domains.

Population-based studies demonstrated significantly higher rates of SC problems in children with mental health conditions. In toddlerhood, deviance in SC seems to be associated with early verbal and non-verbal communication and primarily with internalizing problems such as negative emotionality, social anxiety, inhibition (22, 37), and dysregulation. However, most reviewed studies reported associations of similar strength with all dimensions of mental health, including externalizing (conduct disorder, hyperactivity/attention), internalizing (emotional problems, depressive symptoms, anxiety), and peer-relationship problems. A causal role of SC problems in the emergence or development of mental health conditions cannot be inferred from the cross-sectional studies, as emotional and behavioral disorders are expected to manifest in the way children and adolescents interact with their environment. However, longitudinal population-based studies—mostly based on data from the ALSPAC cohort in the United Kingdom—demonstrated that SC problems at an earlier time point (mostly at preschool and school age before adolescence) are significantly correlated with later mental health symptoms and peer problems in adolescence. In the studies controlling for non-verbal and verbal IQ, structural language, and sex, SC skills still significantly predicted mental health outcomes, showing the specific effect of SC skills. Other longitudinal studies examined SC skills in subgroups defined by the trajectories of mental health symptoms. They demonstrated significantly higher rates of SC difficulties in all studied mental health conditions (depression, ADHD, and conduct problems) in the groups with early onset-persistent and high-severity symptoms, even after controlling for demographic factors and IQ. Accordingly, we can infer that SC skills contribute in a significant and specific way to the emergence and trajectories of mental health problems.

Special-population samples with established or increased vulnerability to mental health problems consistently showed higher rates of SC problems compared to the typically developing controls. In the longitudinal studies of children with developmental language difficulties (49, 55, 56), SC deficits were associated with higher rates of peer problems and emotional problems and reduced prosocial behavior, whereas children with language deficits but no SC problems showed no increase in mental health problems. These findings demonstrate the specific role of SC problems in the pathways to mental health problems. Notably, another longitudinal study (30) of children from a socially disadvantaged background showed that good SC skills at around 8 years of age have a protective role contributing to positive mental health (all SDQ domains) in adolescence.

Even though individuals with ASD were explicitly excluded from this review we are aware of the fact that there is a variation of diagnoses over time (i.e., children with social communication difficulties later diagnosed with ASD or vice versa) and that many of the included studies focus on social communication difficulties rather than clinical diagnoses. Therefore, it can be assumed that some children and adolescents with ASD are still included in the study samples. As a consequence, some of the findings of this review, particularly the role of social communication difficulties for mental health might also be applicable to persons with ASD.

In order to tailor intervention that aims to improve social communication skills in an individual child or adolescent further empirical research and a synthesis of available studies that investigate the dynamic interaction between neurobiological, neuropsychological (e.g., social cognition, social interaction, executive functioning) and environmental factors (e.g., history of maltreatment, quality of parent-child interaction, reactive attachment difficulties) from infancy to young adulthood is required (59–61). The inclusion of pathways to social communication deficits was beyond the scope of this review. However, we certainly need a better understanding of individual pathways to social communication problems for the development and selection of therapeutic approaches.

In summary, SC problems are closely connected with emotional, behavioral, and social problems (particularly peer-relationships) throughout childhood, demonstrated by population-based studies and studies of children with existing or high risk of mental health conditions (research question 1). There is evidence that SC difficulties precede and predict mental health problems and the persistence and severity of symptom trajectories, rather than just following them (research questions 2 and 3). SC difficulties are assumed to negatively affect relationships with peers, the family, and teachers, and consequently to trigger and worsen emotional and behavioral problems. However, these mental health problems might, in turn, lead to withdrawal and lack of opportunities to practice and develop SC skills, representing a vicious circle. The reviewed studies’ results point to mutually reinforcing effects between SC deficits and mental health over time.

Given the unequivocal associations between SC difficulties and mental health problems, greater attention should be given to SC in children with mental health problems and to the mental health of children with communication difficulties. It may be appropriate for mental state examinations to include a stepped approach to SC assessment: brief, age-appropriate symptom checklists; care-giver questionnaires; clinical observations; and standardized assessments. Since SC skills have been shown to moderate mental health trajectories, including preventive effects, interventions to promote SC should be considered as promising in treating mental health disorders. There is growing evidence of the effects of specific training on SC development (6). Furthermore, for a number of psychiatric and neurological conditions [e.g., traumatic brain injury (62), ASD (63, 64)], SC interventions in individual and group therapeutic settings and in everyday social environments shown positive effects.

To introduce SC assessment and intervention in the field of child and adolescent mental health, it is necessary to develop valid and feasible SC measures from infancy to adulthood. Considering the significant role of SC skills in shaping mental health outcomes, we recommend testing available instruments [e.g., the Language Use Inventory (65), CCC-2 (15), and the Brief Observation of Social Communication Change (66)] and direct observations (preferably video-based) of naturally occurring social communication in longitudinal studies of pathways to mental health problems. There is also a need to develop and test the efficacy of manualized intervention programs targeting the specific needs of individuals with diverse mental health problems and associated SC deficits, probably integrated with broader social interventions (67) and other therapy approaches.

## Conclusion

There is robust evidence for significant associations between SC skills and mental health outcomes. Longitudinal studies have demonstrated that SC difficulties can trigger mental health problems and moderate their trajectories. Consequently, we advocate considering the development, evaluation, and systematic introduction of SC assessments and interventions in child and adolescent health and education programs.

## Data availability statement

The original contributions presented in this study are included in the article/Supplementary material, further inquiries can be directed to the corresponding author.

## Author contributions

MD, DH, and JF contributed to the conceptualization and reviewing and final editing of the manuscript. MD and DH contributed to the methodology, data extraction, and writing original draft. MD contributed to the project administration. All authors have read and agreed to the published version of the manuscript.

## Abbreviations

ADHD: attention deficit hyperactivity disorder
ALSPAC: Avon Longitudinal Study of Parents and Children
ASD: autism spectrum disorder
CCC: Children’s Communication Checklist
CCC-2: Children’s Communication Checklist 2nd Edition
CELF-4: Clinical Evaluation of Language Fundamentals 4th Edition
ERRNI: Expression, Reception and Recall of Narratives
IQ: intelligence quotient
NSS: Narrative Scoring Scheme
OR: odds ratio
SC: social communication
SCDC: Social and Communication Disorder Checklist
SCQ: Social Communication Questionnaire
SDQ: Strengths and Difficulties Questionnaire
SIDI: Social Interaction Difference Index.
