# Supplementary material for: The link between social communication and mental health from childhood to young adulthood: A systematic review
Source: Front Psychiatry. 2022 Oct 6;13:944815. doi: 10.3389/fpsyt.2022.944815 (PMC9584641; doi:10.3389/fpsyt.2022.944815)
Supplement: Supplementary file 1 [file Table_1.DOCX]

Supplement 1 Search String for EBSCOhost database (the Psychology and Behavioral Sciences Collection, APA PsychInfo, the Education Resource Information Center (ERIC), and the Cumulative Index to Nursing and Allied Health Literature (CINAHL)) last updated December 23^rd^ 2021

AB ((“social communication” OR “pragmatic language”) AND ((mental OR psychological OR psychosocial OR psychiatric OR behavioral OR behavioural OR emotional) AND (health OR disease OR disorder OR illness OR abnormalit* OR disturbance OR symptom OR problem OR well-being) OR “quality of life” OR anxiety OR depression OR aggression OR “challenging behavior”)) NOT TI autism NOT TI autism spectrum disorders NOT TI autistic
